# miR-200a-3p Attenuates Coronary Microembolization-Induced Myocardial Injury in Rats by Inhibiting TXNIP/NLRP3-Mediated Cardiomyocyte Pyroptosis

**DOI:** 10.3389/fcvm.2021.693257

**Published:** 2021-08-05

**Authors:** Zhi-Qing Chen, You Zhou, Feng Chen, Jun-Wen Huang, Hao-Liang Li, Tao Li, Lang Li

**Affiliations:** ^1^Department of Cardiology, The First Affiliated Hospital of Guangxi Medical University, Nanning, China; ^2^Department of Emergency, The First Affiliated Hospital of Guangxi Medical University, Nanning, China

**Keywords:** miR-200a-3p, coronary microembolization, myocardial injury, pyroptosis, TXNIP/NLRP3 signaling pathway

## Abstract

Coronary microembolization (CME) commonly develops as a complication after percutaneous coronary intervention (PCI), and associated inflammation is a leading driver of myocardial damage. Cardiomyocyte loss in the context of ischemic myocardial disease has been linked to inflammatory pyroptotic cell death. Additionally, miR-200a-3p dysregulation has been linked to myocardial ischemia-reperfusion and many other pathological conditions. However, how miR-200a-3p impacts cardiomyocyte pyroptosis in the context of CME remains to be assessed. Herein, a rat model of CME was established via the injection of microembolic spheres into the left ventricle. When myocardial tissue samples from these rats were analyzed, miR-200a-3p levels were markedly decreased, whereas thioredoxin-interacting protein (TXNIP) levels were increased. The ability of miR-200a-3p to directly target TXNIP and to control its expression was confirmed via dual-luciferase reporter assay. Adeno-associated virus serotype 9-pre-miR-200a-3p (AAV-miR-200a-3p) construct transfection was then employed as a means of upregulating this miRNA in CME model rats. Subsequent assays, including echocardiography, enzyme-linked immunosorbent assays (ELISAs), hematoxylin-eosin (H&E) staining, hematoxylin-basic fuchsin-picric acid (HBFP) staining, TdT-mediated dUTP nick-end labeling (TUNEL) staining, immunofluorescence staining, quantitative real-time polymerase chain reaction (qRT-PCR), and Western blotting revealed that miR-200a-3p overexpression inhibited cardiomyocyte pyroptosis and alleviated CME-induced myocardial injury by inhibiting the TXNIP/NOD-like receptor family pyrin domain-containing 3 (NLRP3) pathway. The ability of miR-200a-3p to protect against CME-induced myocardial injury thus highlights a novel approach to preventing or treating such myocardial damage in clinical settings.

## Introduction

Coronary microembolization (CME) is a serious complication of percutaneous coronary intervention (PCI) that is independently predictive of negative cardiac events and poor long-term patient outcomes in the context of the slow- or no-reflow phenomenon (1). CME is characterized by the formation of microemboli and microinfarcts within the coronary microvasculature and myocardial tissue as a consequence of the occlusion of coronary microvessels caused by spontaneous atherosclerotic plaque rupture or plaque fragments (2). The acute phase of CME can drive myocardial inflammation, apoptotic cell death, and necrosis, with subsequent cardiac systolic dysfunction and myocardial injury being promoted in large part by these inflammatory processes (3, 4).

Pyroptosis is a form of inflammatory cell death that is physiologically distinct from apoptosis or necrosis, and that is driven by pro-inflammatory factors and the activation of the NOD-like receptor family pyrin domain-containing 3 (NLRP3)/caspase-1 signaling pathway (5). The NLRP3 inflammasome is composed of ASC and caspase-1 (6), and the activation of NLRP3 in the context of pyroptosis induces ASC-mediated caspase-1 activation, which subsequently cleaves the pro-forms of interleukin-1β (IL-1β)/IL-18 to generate mature versions of these molecules that can be released from cells (7). Gasdermin D (GSDMD) can additionally be cleaved by caspase-1, resulting in the release of its N-terminal pore-forming domain (GSDMD-N), which is subsequently inserted into cellular membranes to facilitate inflammatory factor release, cellular swelling, and membrane rupture (8). Pyroptosis has previously been linked to myocardial infarction, myocardial ischemia-reperfusion injury, diabetic cardiomyopathy, and heart failure (9), but its mechanistic role in the context of CME remains to be defined and thus warrants further study in an effort to define novel approaches to preventing CME-induced myocardial damage.

The NLRP3 inflammasome is a key mediator of diverse inflammatory processes (10), and we have previously demonstrated its activation in the context of CME wherein it is was associated with myocardial inflammation, cardiac dysfunction, and myocardial damage (11). Thioredoxin-interacting protein (TXNIP) is an inhibitor of thioredoxin that controls oxidative homeostasis within cells and that regulates NLRP3 inflammasome activation in the context of pyroptosis (12). In studies of intestinal ischemia-reperfusion injury, TXNIP has been shown to directly activate the NLRP3 inflammasome to induce pyroptotic cell death (13), whereas taurine has been shown to inhibit this TXNIP/NLRP3 inflammatory pathway and to thereby disrupt downstream inflammation to protect against Schistosomiasis japonicum-induced hepatic injury (14). This TXNIP/NLRP3 inflammatory pathway is thus likely to be a central mediator of pyroptosis, but how it impacts CME remains to be evaluated.

MicroRNAs (miRNAs) are short (~22 nucleotide) RNAs lacking coding potential that are able to control essential processes including proliferation, differentiation, and cell survival (15). These miRNAs primarily function by binding to the 3′-untranslated region (UTR) of target mRNAs and thereupon driving mRNA degradation or translational inhibition (16). Several miRNAs have been identified as important regulators of myocardial infarction, heart failure, and hypertrophic cardiomyopathy (17). For example, miR-21 can target kelch repeat and BTB (POZ) domain containing 7 to suppress p38 and NF-κB signaling, thereby protecting against myocardial infarction-associated inflammation and cardiac dysfunction (18). Lu et al. found miR-134-5p downregulation to protect against hypoxia/reoxygenation-induced cardiomyocyte injury by suppressing oxidative stress and apoptotic signaling (19). Importantly, miR-26a-5p has been shown to suppress CME-induced myocardial injury in rats by modulating HMGA1 expression to suppress myocardial inflammation, thereby decreasing the microinfarct area and ameliorating cardiac function (20). In several previous analyses, miR-200a-3p has been linked to a range of diseases including gastric cancer (21), ovarian cancer (22), and Alzheimer's disease (23). In myocardial cells, miR-200a-3p can suppress PDCD4 expression in hypoxic contexts to protect against apoptosis (24). Notably, a previous study reported that miR-200a degradation in a rat model of diabetic nephropathy can lead to renal damage by stimulating pyroptosis via the TXINP/NLRP3 signaling pathway (25). The interaction between miR-200a-3p and TXNIP in the context of CME and its effect on CME-induced myocardial injury, however, remains to be established.

This study was the first to our knowledge to have explored the mechanistic importance of miR-200a-3p in the context of CME-induced myocardial injury. Through these analyses, we determined that miR-200a-3p can reduce the severity of CME-induced myocardial pyroptosis by suppressing TXNIP/NLRP3 pathway activation, highlighting a promising new approach to the therapeutic prevention or treatment of CME-induced myocardial injury.

## Materials and Methods

### Animal Care

Sprague Dawley (SD) rats (8 weeks old, 250–300 g) from the Experimental Animal Center of Guangxi Medical University were housed in a climate-controlled facility (23–25°C) with free food and water access. The Animal Use Ethics Committee of Guangxi Medical University approved the present study, which was consistent with the Guidelines for the Care and Use of Laboratory Animals.

### Experimental Design

To explore the expression of miR-200a-3p and TXNIP in the context of CME, SD rats were randomized into a sham group and a CME group (*n* = 10/group). For studies of how miR-200a-3p and TXNIP impact CME, rats were randomized into sham, CME, CME + adeno-associated virus serotype 9 (AAV)-negative control (CME + AAV-NC) and CME + AAV-pre-miR-200a-3p (CME + AAV-miR-200a-3p) groups (*n* = 10/group). AAV-miR-200a-3p or AAV-NC at a dose of 1 × 10^12^ vector genomes (v.g.) was injected into rats via the tail vein 4 weeks prior to CME modeling. AAV-miR-200a-3p and AAV-NC were designed and synthesized by Shanghai Genechem Co., Ltd (Shanghai, China).

### CME Rat Modeling

A rat model of CME was established as published previously (26). Briefly, rats were anesthetized via intraperitoneally injecting 30–40 mg/kg pentobarbital sodium, after which an endotracheal tube connected to a ventilator was inserted via the mouth of each rat to support respiration. Layer-by-layer thoracotomy in the second to fourth intercostal space was then conducted on the left edge of the sternum until the heart and associated vasculature were clearly visible. A vascular clamp was then used to occlude the ascending aorta for 10 s, while 4,000 microspheres (45 μm diameter, Polysciences Inc., USA) in 0.2 mL of normal saline were injected into the left ventricle from the apex of the heart. Sham controls were instead injected with normal saline free of microspheres. Following the restoration of a normal heart rate and spontaneous breathing, the chest was closed in a layer-by-layer manner, and extubation was then performed. At 12 h postoperatively, echocardiography was performed and blood samples were collected, after which rats were euthanized and myocardial samples were isolated for downstream analysis.

### Echocardiography

Prior reports suggest that rat cardiac function reaches a minimum level at 12 h post-CME (27), and this time point was therefore used in the present study. A Philips Sonos 7,500 ultrasound system (Philips Technologies, MA, USA) with a 12 MHz probe was used for echocardiographic analyses of left ventricular ejection fraction (LVEF), left ventricular fractional shortening (LVFS), left ventricular end-diastolic diameter (LVEDd), and left ventricular end-systolic diameter (LVESd). A specialist with extensive echocardiography experience made all measurements.

### Myocardial Injury, Inflammatory, and Oxidative Stress Analyses

Aortic blood samples were obtained at 12 h post-model establishment. Levels of serum cardiac troponin I (cTnI), IL-18, and IL-1β were assessed with a commercial enzyme-linked immunosorbent assay (ELISA) kit (Bio-Swamp Biological Technology Co., Ltd., Wuhan, China), while serum lactate dehydrogenase (LDH) and creatine kinase myocardial band isoenzyme (CK-MB) levels were analyzed with an automatic biochemical analyzer (HITACHI 7600-020, Japan). Levels of malondialdehyde (MDA) and superoxide dismutase (SOD) in myocardial tissue samples were assessed with appropriate kits (Nanjing Jiancheng Bio-Technology co., Ltd, Nanjing, China) based upon provided directions.

### Sample Collection

Following the completion of the above steps, rats were euthanized via the intravenous injection of 10% potassium chloride (2 mL). Cardiac tissues were then isolated, rinsed with chilled normal saline, and separated into apical sections that were respectively stored at −80°C for Western blotting and quantitative real-time polymerase chain reaction (qRT-PCR), and basal sections that were fixed for 12 h with 4% paraformaldehyde, paraffin-embedded, and utilized for downstream hematoxylin-eosin (H&E), hematoxylin-basic fuchsin-picric acid (HBFP), TdT-mediated dUTP nick-end labeling (TUNEL), and immunofluorescence staining.

### H&E Staining

Myocardial histopathological changes were evaluated via H&E staining. Briefly, paraffin-embedded tissue sections were sliced into 4 μm sections, deparaffinized using xylene, and stained for 3 min with hematoxylin followed by a 3 min counterstain with eosin. Sections were then analyzed via light microscope (Olympus, Tokyo, Japan).

### Myocardial Infarct Size Measurement

Early myocardial ischemia was detected via HBFP staining (28), wherein normal cardiomyocytes exhibit blue nuclei and yellow cytoplasms while ischemic cardiomyocytes and erythrocytes are red. Each field of view (×200) was assessed via light microscopy (Olympus, Tokyo, Japan), with Image-Pro Plus 6.0 (Media Cybernetics, MD, USA) being used to measure infarct size as follows: microinfarct area / total analysis area ×100%.

### TUNEL Staining

Using methods detailed in prior studies (25, 29), cardiomyocyte death was evaluated with a TUNEL assay kit (Roche, USA) based on provided directions in order to reflect the degree of pyroptosis in myocardial tissues. Under an optical microscope (Olympus, Tokyo, Japan), nuclei of normal and apoptotic cells appeared light blue and yellow-brown after staining, respectively. The apoptotic index value was calculated as the number of apoptotic cells divided by the total number of cells in each field of view (×400) (30).

### Immunofluorescence Staining

Myocardial TXNIP, NLRP3, cleaved caspase-1, and GSDMD-N expression were assessed via immunofluorescence staining. Briefly, frozen tissue sections were washed thrice with PBS (5 min/wash), after which they were permeabilized for 30 min using 0.3% Triton X-100 in TBS, blocked for 30 min with goat serum at 37°C, washed thrice with PBS (5 min/wash), and incubated overnight with primary antibodies specific for TXNIP, NLRP3, GSDMD-N, or cleaved caspase-1 at 4°C. After three further washes, sections were probed using Cy3-conjugated secondary antibodies (Abcam, USA) at 37°C for 1 h. DAPI in anti-fading sealant (Servicebio technology Co., Ltd., Wuhan, China) was then used to counterstain nuclei, and samples were assessed via fluorescence microscope (Olympus, Tokyo, Japan).

### qRT-PCR

Trizol (Invitrogen, USA) was used to extract RNA from samples, after which RNA concentrations were assessed with a NanoDrop 2000 instrument (Thermo Fisher Scientific Inc., USA). Next, cDNA was prepared from RNA samples, and qRT-PCR was conducted using an ABI PRISM 7500 instrument (Applied BioSystems, USA) and a SYBR Green I kit (TaKaRa, Tokyo, Japan). Each reaction was 20 μL in volume, and samples were assessed in triplicate. U6 and GAPDH were respectively used to normalize miR-200a-3p and mRNA expression. Relative gene expression was assessed via the 2^−Δ*ΔCt*^ approach. Primers used in this assay are shown in Table 1.

**Table 1:** Primer sequence.

**Gene**	**Primer sequence**
miR-200a-3p	Forward: 5′-GGCACTGTCTGGTAACGATGTAA-3′ Reverse: 5′-TGGTGTCGTGGAGTCG-3′
U6	Forward: 5′-CTCGCTTCGGCAGCACA-3′ Reverse: 5′-TGGTGTCGTGGAGTCG-3′
TXNIP	Forward: 5′-CTGGGTGAAGGCTTTTCTCG-3′ Reverse: 5′-CTCAAAGTCAGCGTGGATGG-3′
NLRP3	Forward: 5′-CTCGCATTGGTTCTGAGCTC-3′ Reverse: 5′-AGTAAGGCCGGAATTCACCA-3′
ASC	Forward: 5′-GCTGAGCAGCTGCAAAAGAT-3′ Reverse: 5′-GCAATGAGTGCTTGCCTGTG-3′
IL-18	Forward: 5′-TGCTCATCATGCTGTTCTGC-3′ Reverse: 5′-AGCCAAGAATCTCCGTAGCA-3′
IL-1β	Forward: 5′-GGGATGATGACGACCTGCTA-3′ Reverse: 5′-TGTCGTTGCTTGTCTCTCCT-3′
GAPDH	Forward: 5′-TTTGAGGGTGCAGCGAACTT-3′ Reverse: 5′-ACAGCAACAGGGTGGTGGAC-3′

### Western Blotting

Cold RIPA buffer supplemented with protease inhibitors was used to lyse homogenized myocardial tissue samples, after which a bicinchoninic acid (BCA) assay (Beyotime, Shanghai, China) was used to quantify protein concentrations in these lysates. Protein samples were then separated via 10% SDS-PAGE and transferred onto PVDF membranes (Millipore, MA, USA). Blots were then blocked for 1 h using 5% non-fat milk, after which they were incubated overnight with primary antibodies specific for TXNIP, NLRP3, ASC, pro-caspase-1, cleaved caspase-1, GSDMD-FL, GSDMD-N, IL-1β, IL-18, and β-actin at 4°C. The specific primary antibodies against pro-caspase-1 and cleaved caspase-1 were diluted at 1:500, and all other antibodies were diluted at 1:1000. Antibodies specific for IL-18, and IL-1β, ASC, NLRP3, and TXNIP were from Abcam (MA, USA), antibodies specific for pro-caspase-1 and cleaved caspase-1 were obtained from Santa Cruz Biotechnology (TX, USA), and antibodies specific for GSDMD-FL, GSDMD-N, and β-actin were purchased from Cell Signaling Technology (MA, USA). Blots were then washed thrice with TBST (5 min/wash), followed by incubation with HRP-conjugated secondary antibodies (Santa Cruz Biotechnology, TX, USA) for 2 h at room temperature. After three additional washes, an enhanced chemiluminescence (ECL) reagent was used to detect protein bands. β-actin was used as a loading control, while densitometric analyses were conducted using ImageJ (National Institutes of Health, MD, USA).

### Dual-Luciferase Reporter Assay

The ability of miR-200a-3p to interact with the TXNIP 3′-untranslated region (UTR) was predicted using the TargetScan (http://www.targetscan.org) and miRDB (http://mirdb.org) databases. These putative interactions were then tested via a luciferase reporter approach. Briefly, wild-type (wt) and mutant (mut) versions of the TXNIP 3′-UTR were cloned into the psiCHECK-2 vector (Hanbio Biotechnology Co., Ltd., Shanghai, China) to prepare reporter plasmids which were then co-transfected into HEK-293T cells along with miR-200a-3p mimics or corresponding negative control constructs using Lipofectamine 2000 (Invitrogen, USA). At 48 h post-transfection, a Dual-Luciferase assay kit (Promega, USA) was used based on provided directions, with Renilla luciferase being used for normalization purposes.

### Statistical Analysis

All data were expressed as mean ± standard deviation (SD), and were analyzed with SPSS 17.0 (SPSS, Inc., IL, USA). Data were compared via Student's *t*-tests or one-way analysis of variance (ANOVA) with the Bonferroni *post hoc* test as appropriate. *P* < 0.05 was the threshold of significance. Figures were prepared using Graphpad Prism 8.0 (GraphPad Software, CA, USA).

## Results

### Development of a Rat Model of CME-Induced Myocardial Injury

**Figure 1:**
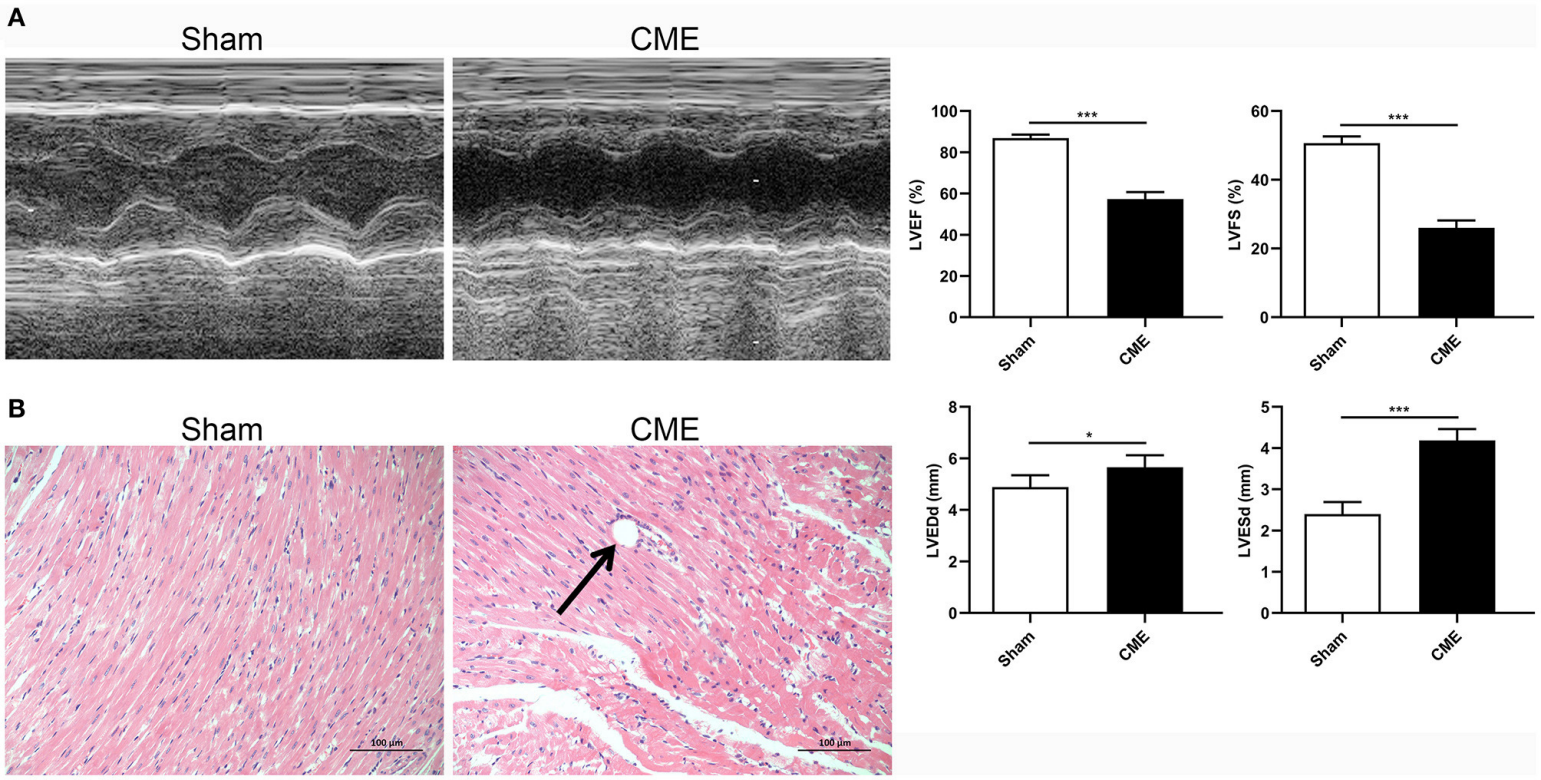
CME induced myocardial injury in rats. **(A)** Echocardiographic analysis of cardiac function (*n* = 6 per group), and corresponding comparison of left ventricle ejection fraction (LVEF), left ventricle fractional shortening (LVFS), left ventricular end-diastolic diameter (LVEDd), and left ventricular end-systolic diameter (LVESd). **(B)** Histopathological analysis by H&E staining (×200 magnification; bar = 100 μm) (*n* = 3 per group). The data are presented as the mean ± standard deviation (SD). ^*^P < 0.05, ^***^P < 0.001. CME, coronary microembolization.

To demonstrate the effects of CME on the rat myocardium, we began by assessing cardiac contractile function indices and corresponding pathological changes in our model rats. Rats in the CME group exhibited significantly reduced systolic function relative to sham controls, as evidenced by reduced LVEF and LVFS and increased LVEDd and LVESd (*P* < 0.05, Figure 1A). H&E staining (Figure 1B) revealed no apparent pathological changes in the myocardial tissue of sham group, whereas those in the CME group exhibited small arterial microembolic spheres, with cardiomyocyte nuclei present in the center of these embolic foci being absent or dissolved and with adjacent cells appearing swollen. Inflammatory cell infiltration and erythrocyte exudation were also evident in the peripheral infarct area. These findings indicated that our CME model had been established successfully and that it induced myocardial injury in experimental rats.

### CME Induces miR-200a-3p Downregulation and Pyroptosis in Rat Myocardial Tissues

**Figure 2:**
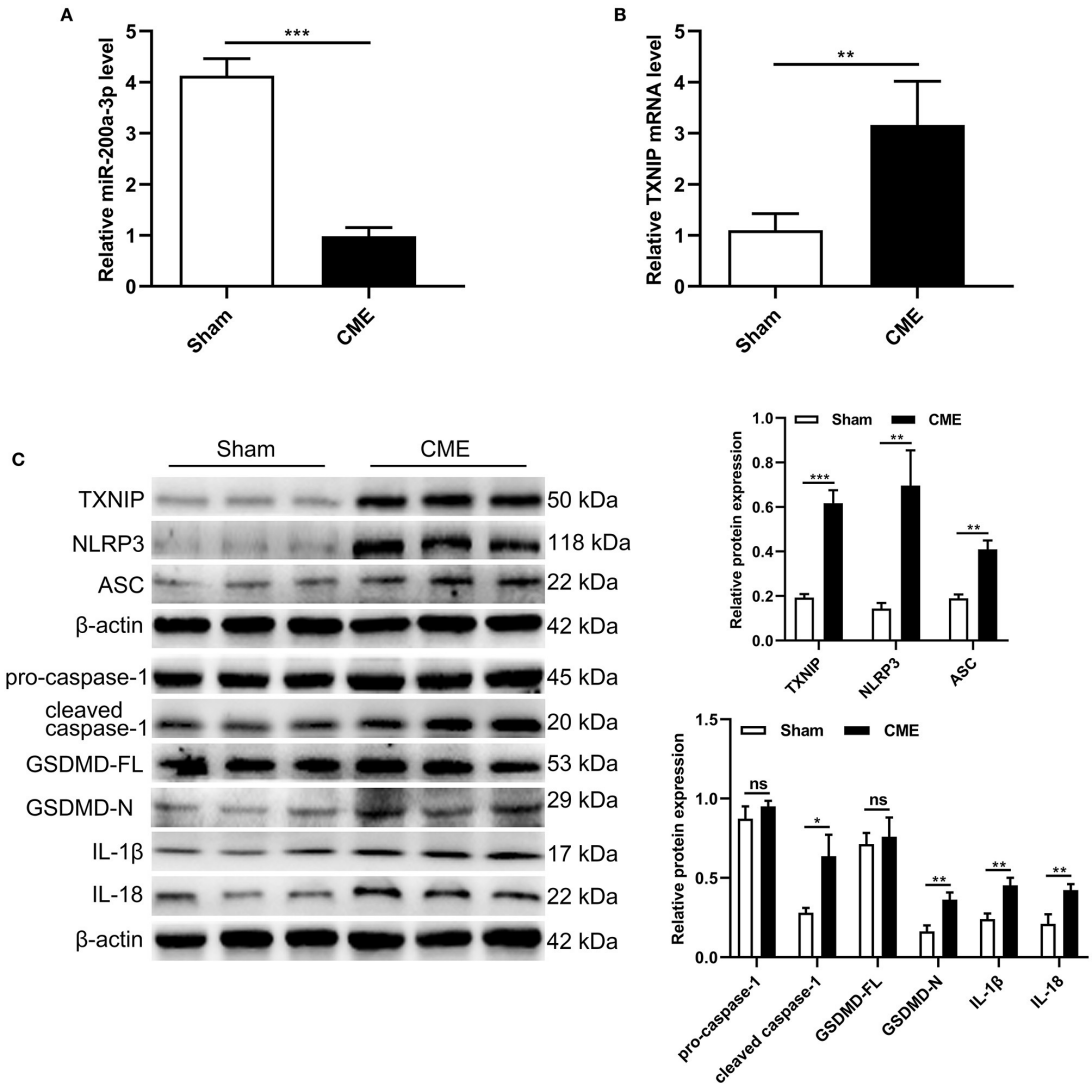
CME induces miR-200a-3p downregulation and pyroptosis in rat myocardial tissues. **(A)** Relative miR-200a-3p expression level was assessed via qRT-PCR (*n* = 6 per group). **(B)** Relative TXNIP mRNA expression level was assessed via qRT-PCR (*n* = 5 per group). **(C)** Expression levels of proteins related to cardiomyocyte pyroptosis (*n* = 3 per group). The data are presented as the mean ± standard deviation (SD). ^*^P < 0.05, ^**^P < 0.01, ^***^P < 0.001, ns, not significant. CME, coronary microembolization.

To assess how CME impacts the expression of miR-200a-3p and TXNIP and the induction of pyroptotic cell death, we next assessed myocardial miR-200a-3p and TXNIP mRNA levels via qRT-PCR and the expression of pyroptosis-associated proteins via Western blotting. CME model rats exhibited significant miR-200a-3p downregulation and TXNIP upregulation relative to sham controls (*P* < 0.05, Figures 2A,B). Moreover, TXNIP, NLRP3, ASC, cleaved caspase-1, GSDMD-N, IL-1β, and IL-18 protein levels were significantly increased in CME model rats relative to controls (*P* < 0.05, Figure 2C), whereas no differences in pro-caspase-1 or GSDMD-FL levels were observed between these groups (*P* > 0.05). These data suggest that CME can promote the downregulation of miR-200a-3p, the upregulation of TXNIP, and the induction of pyroptotic cell death.

### miR-200a-3p Targets TXNIP to Suppress Its Expression

**Figure 3:**
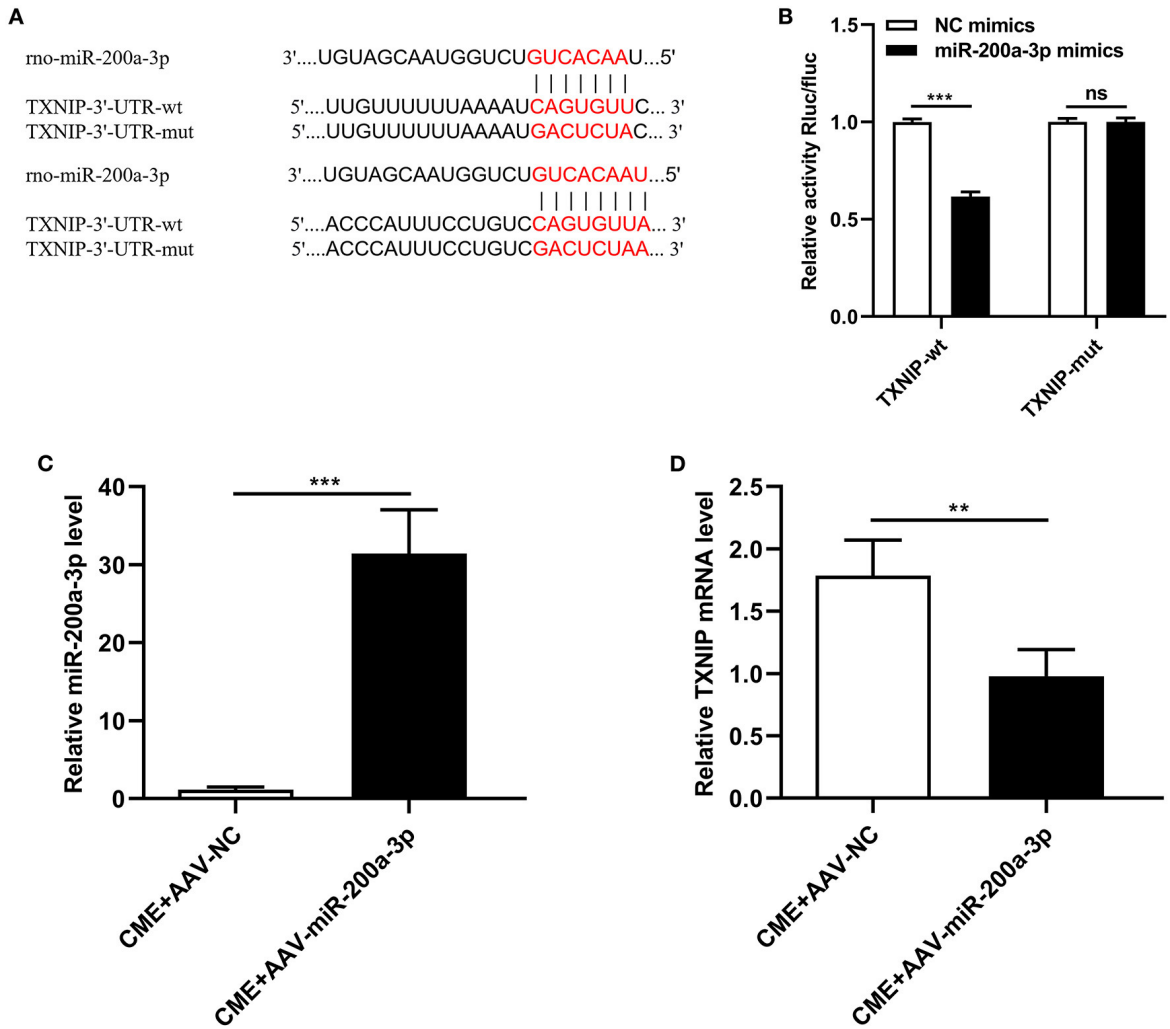
miR-200a-3p directly suppresses TXNIP expression. **(A)** Schematic of complementary miR-200a-3p and TXNIP 3′-UTR sequences. **(B)** HEK-293T cells were co-transfected with wild-type or mutant TXNIP 3′-UTR reporter constructs and miR-200a-3p mimics or corresponding negative controls. **(C)** qRT-PCR was used to assess myocardial miR-200a-3p levels (*n* = 4 per group). **(D)** qRT-PCR was used to assess myocardial TXNIP mRNA levels (*n* = 5 per group). The data are presented as the mean ± standard deviation (SD). ^**^P < 0.01, ^***^P < 0.001, ns, not significant. CME, coronary microembolization. wt, wild-type; mut, mutant; UTR, untranslated region.

Next, a dual-luciferase reporter assay approach and qRT-PCR were used to test the association between miR-200a-3p and TXNIP. We found that the TXNIP 3′-UTR contained putative binding sites for miR-200a-3p (Figure 3A). Luciferase reporter assays confirmed that the luciferase activity was markedly reduced when cells were transfected with miR-200a-3p mimics when using the wt-TXNIP reporter (*P* < 0.05, Figure 3B), whereas no significant change was observed for the mut-TXNIP reporter (*P* > 0.05). Furthermore, following AAV-miR-200a-3p administration in the rat model of CME, rat myocardial tissues exhibited significant increases in miR-200a-3p expression and significant decreases in TXNIP mRNA levels (*P* < 0.05, Figures 3C,D). These data suggest that miR-200a-3p can directly target TXNIP to suppress its expression in myocardial tissues.

### miR-200a-3p Upregulation Alleviates CME-Induced Myocardial Injury

**Figure 4:**
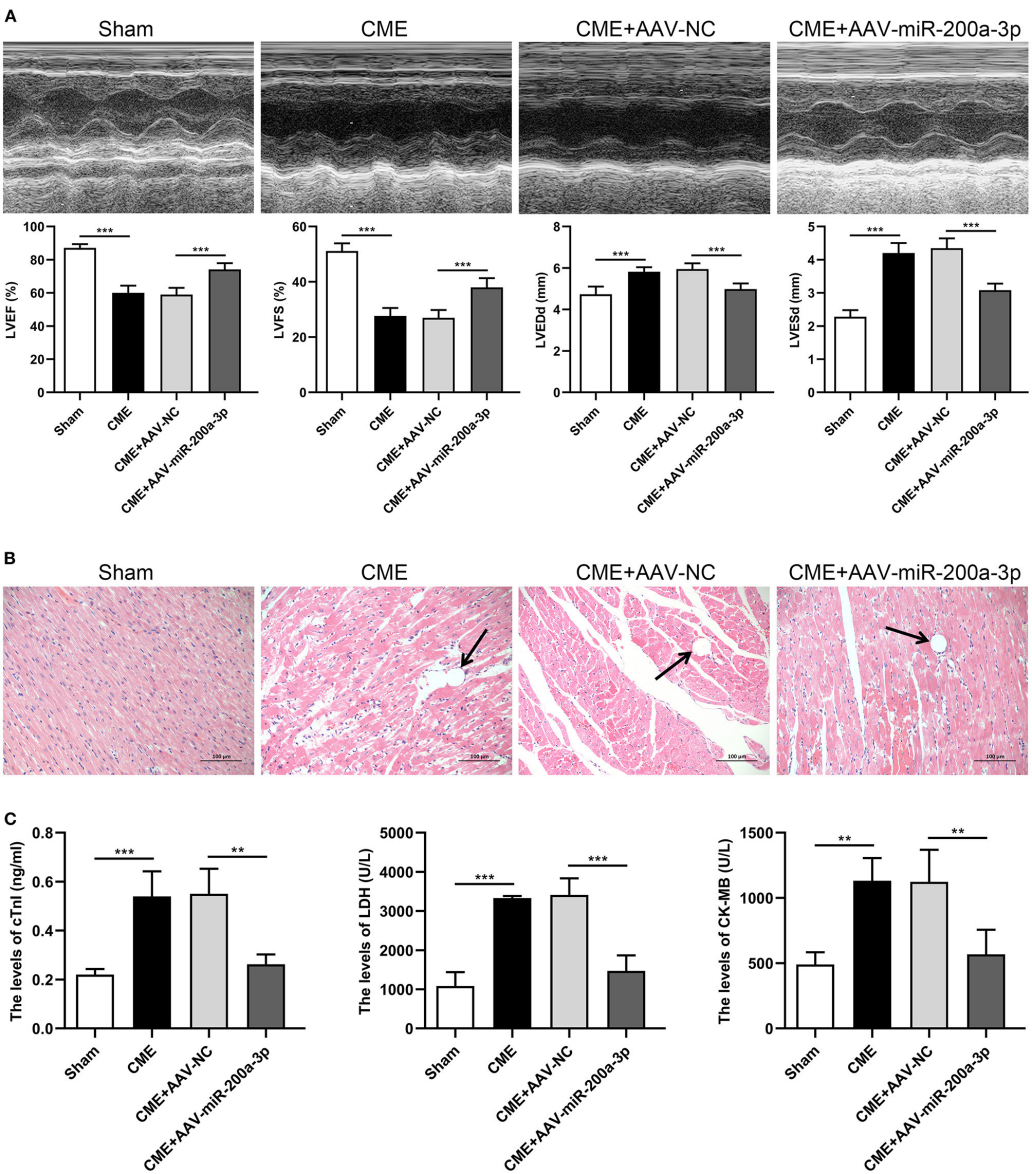
miR-200a-3p overexpression alleviates CME-induced myocardial injury. **(A)** Echocardiographic analysis of cardiac function in each group (*n* = 6 per group), and corresponding comparison of left ventricle ejection fraction (LVEF), left ventricle fractional shortening (LVFS), left ventricular end-diastolic diameter (LVEDd), and left ventricular end-systolic diameter (LVESd). **(B)** Histopathological analysis in each group by H&E staining (×200 magnification; bar = 100 μm) (*n* = 3 per group). **(C)** The serum levels of cTnI, LDH, and CK-MB in each group (*n* = 4 per group). The data are presented as the mean ± standard deviation (SD). ^**^P < 0.01, ^***^P < 0.001. CME, coronary microembolization.

To assess how miR-200a-3p expression is associated with CME-induced myocardial injury, we administered AAV-miR-200a-3p to rats in order to upregulate this miRNA, and we then evaluated cardiac contractile function indices, cardiomyocyte pathological changes, and serum myocardial injury marker levels in these animals. Relative to rats in the CME+AAV-NC group, those in the CME+AAV-miR-200a-3p group exhibited significantly improved cardiac contractility as evidenced by increases in LVEF and LVFS and by decreases in LVEDd and LVESd (*P* < 0.05, Figure 4A). H&E staining indicated that miR-200a-3p upregulation was associated with reduced myocardial injury relative to control treatment (Figure 4B). Additionally, serum cTnI, LDH, and CK-MB levels were notably increased in CME model animals relative to the sham group. The overexpression of miR-200a-3p significantly reduced serum cTnI, LDH, and CK-MB levels compared to the CME+AAV-NC group (*P* < 0.05, Figure 4C). Our findings thus suggested that miR-200a-3p upregulation can reduce the severity of myocardial injury and improve cardiac function in CME model rats.

### miR-200a-3p Upregulation Reduces CME-Induced Myocardial Microinfarct Size and Cardiomyocyte Death

**Figure 5:**
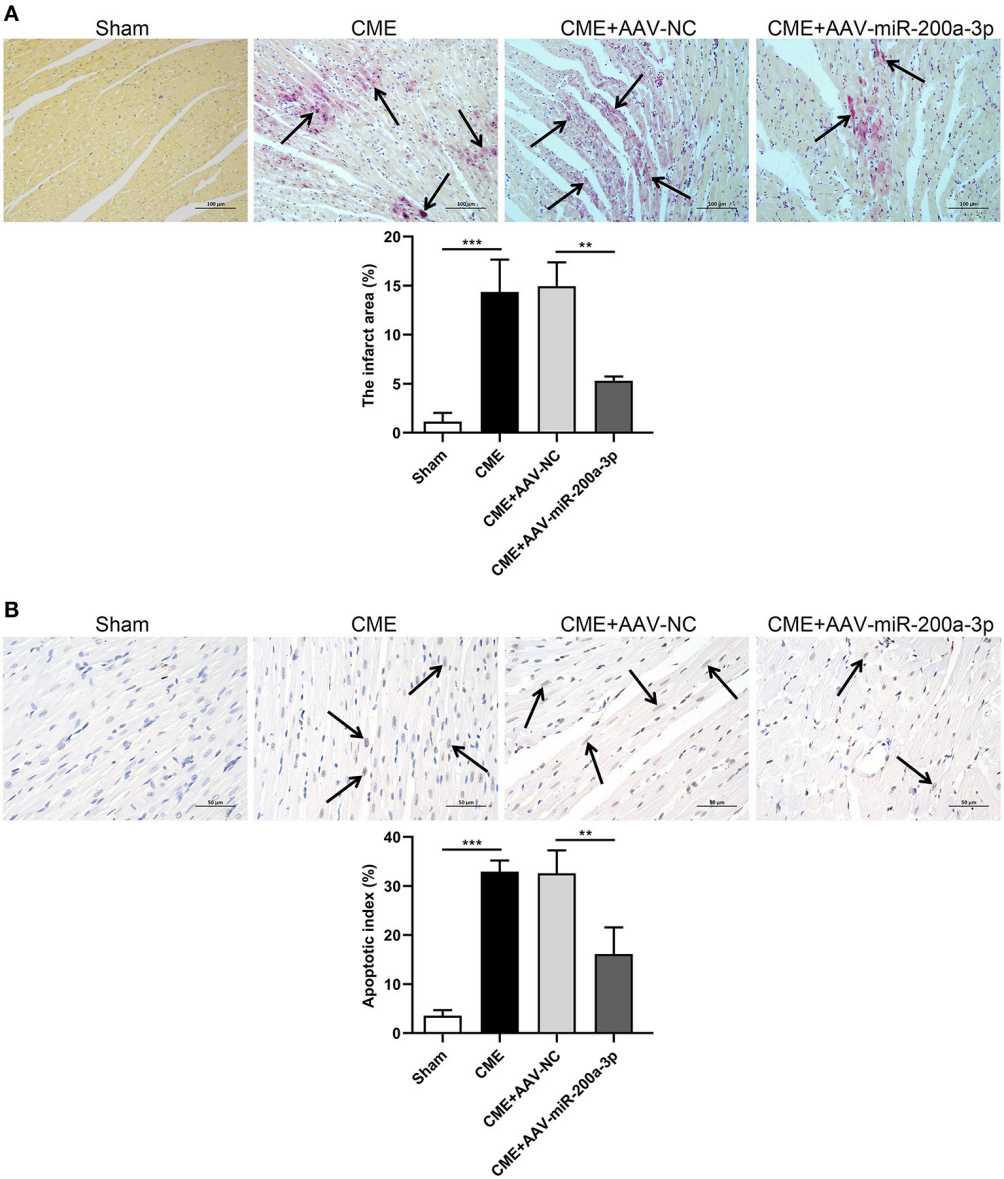
miR-200a-3p overexpression decreases CME-induced myocardial microinfarct size and alleviates cardiomyocyte death. **(A)** HBFP staining (×200 magnification; bar = 100 μm) was used to detect normal (yellow) and ischemic (red) cardiomyocytes in myocardial tissues. Arrows are used to denote the microinfarct area (*n* = 3 per group). **(B)** TUNEL staining (×400 magnification; bar = 50 μm) was used to detect normal (light blue) and apoptotic (yellow-brown) cardiomyocytes in myocardial tissues. Arrows are used to denote apoptotic cardiomyocyte nuclei (*n* = 3 per group). The data are presented as the mean ± standard deviation (SD). ^**^P < 0.01, ^***^P < 0.001. CME, coronary microembolization.

As a means of exploring the effects of miR-200a-3p overexpression on the CME-induced myocardial microinfarct area and cardiomyocyte death, we conducted HBFP and TUNEL staining, respectively. HBFP staining indicated that sham control rats were free of any microinfarcts, whereas rats in the CME and CME+AAV-NC groups exhibited significant microinfarct formation. However, CME+AAV-miR-200a-3p treatment was associated with a significant reduction in microinfarct size relative to control CME+AAV-NC treatment (*P* < 0.05, Figure 5A). The microinfarct areas in sham, CME, CME+AAV-NC and CME+AAV-miR-200a-3p groups were 1.13 ± 0.90%, 14.36 ± 3.30%, 14.95 ± 2.42%, and 5.30 ± 0.42%, respectively. TUNEL staining further revealed that sham control rats exhibited relatively few apoptotic cardiomyocytes, whereas large numbers of apoptotic cardiomyocytes were detected in the microinfarct region and surrounding regions in rats in the CME and CME+AAV-NC groups. However, rats in the CME+AAV-miR-200a-3p group exhibited significantly fewer apoptotic cardiomyocytes relative to the CME+AAV-NC group (*P* < 0.05, Figure 5B). The rates of cardiomyocyte apoptosis in the sham, CME, CME+AAV-NC and CME+AAV-miR-200a-3p groups were 3.55 ± 1.15%, 32.96 ± 2.25%, 32.60 ± 4.68%, and 16.14 ± 5.44%, respectively. Our findings suggest that miR-200a-3p upregulation can decrease myocardial microinfarct size and inhibit cardiomyocyte death in CME model rats.

### miR-200a-3p Upregulation Suppresses CME-Induced Oxidative Stress

**Figure 6:**
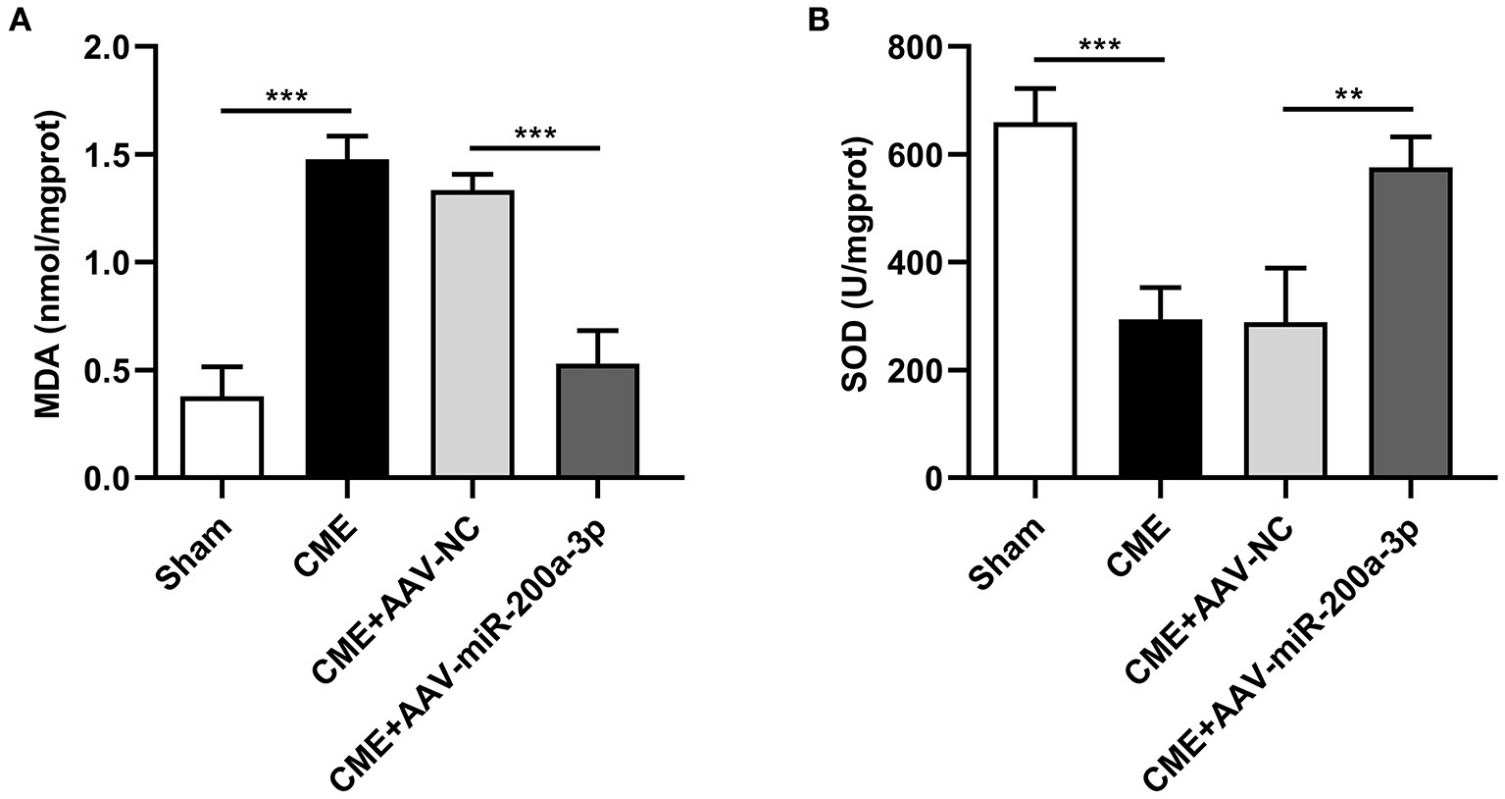
miR-200a-3p overexpression suppresses CME-induced oxidative stress. **(A)** Myocardial MDA levels were assessed in each group (*n* = 4 per group). **(B)** Myocardial SOD levels were assessed in each group (*n* = 4 per group). The data are presented as the mean ± standard deviation (SD). ^**^P < 0.01, ^***^P < 0.001. CME, coronary microembolization.

To evaluate the impact of miR-200a-3p expression on CME-induced oxidative stress, we next assessed myocardial MDA and SOD levels of rats in each group (Figures 6A,B). Compared to the sham group, MDA levels were increased in the CME group, whereas SOD levels therein were significantly decreased. In contrast, MDA levels were significantly reduced in rats in the CME+AAV-miR-200a-3p group relative to those in the CME+AAV-NC group, while SOD levels were significantly increased in these rats. These data suggest that miR-200a-3p upregulation inhibits CME-induced oxidative stress.

### miR-200a-3p Upregulation Inhibits TXNIP/NLRP3 Signaling Pathway to Alleviate CME-Induced Cardiomyocyte Pyroptosis

**Figure 7:**
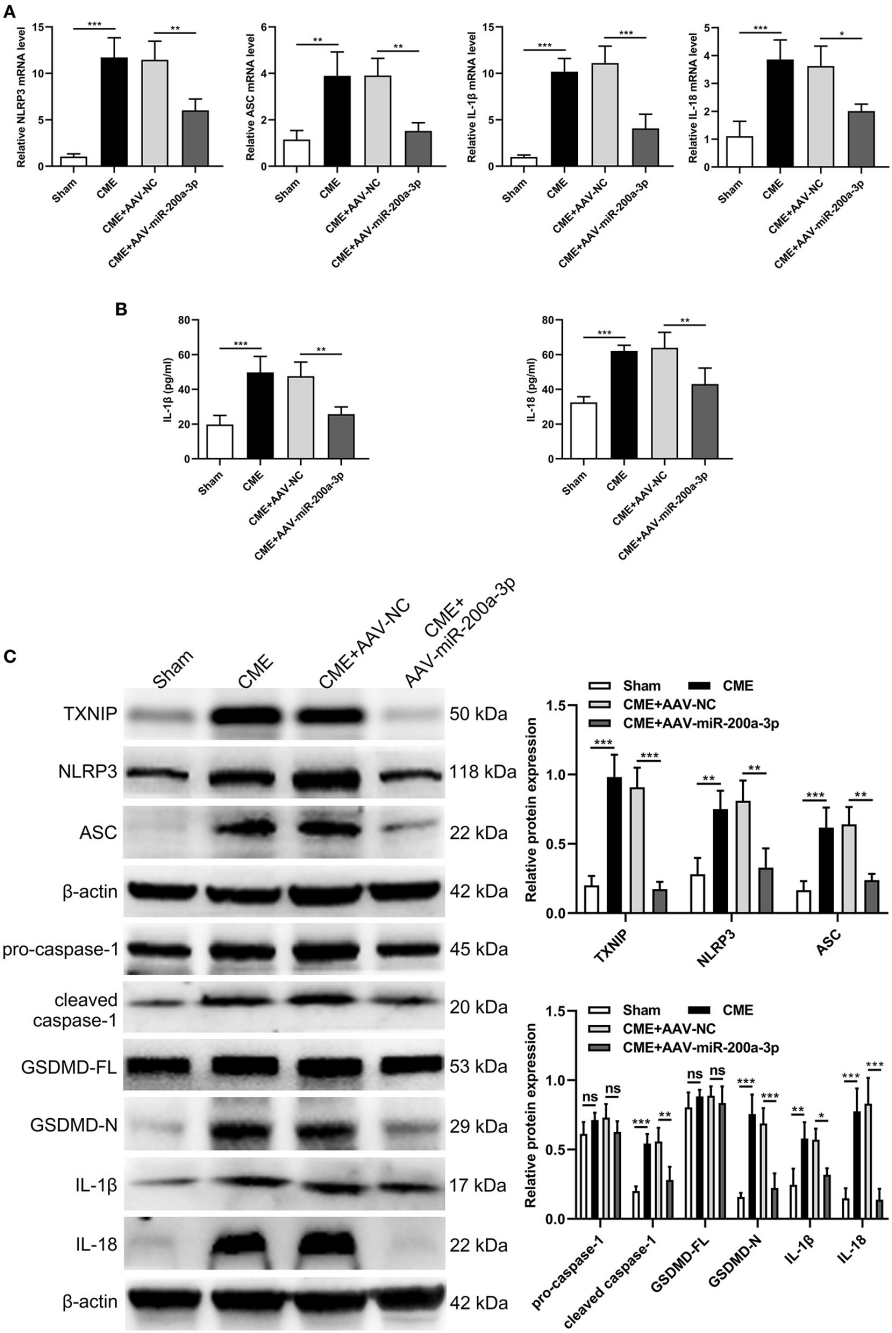
miR-200a-3p overexpression decreases CME-induced cardiomyocyte pyroptosis via the inhibition of the TXNIP/NLRP3 pathway. **(A)** Myocardial NLRP3, ASC, IL-1β, and IL-18 mRNA expression levels were assessed via qRT-PCR (*n* = 4 per group). **(B)** Serum IL-1β and IL-18 expression levels were assessed via ELISA (*n* = 4 per group). **(C)** Myocardial TXNIP, NLRP3, ASC, pro-caspase-1, cleaved caspase-1, GSDMD-FL, GSDMD-N, IL-1β, and IL-18 protein levels were assessed via Western blotting (*n* = 4 per group). The data are presented as the mean ± standard deviation (SD). ^*^P < 0.05, ^**^P < 0.01, ^***^P < 0.001, ns, not significant. CME, coronary microembolization.

**Figure 8:**
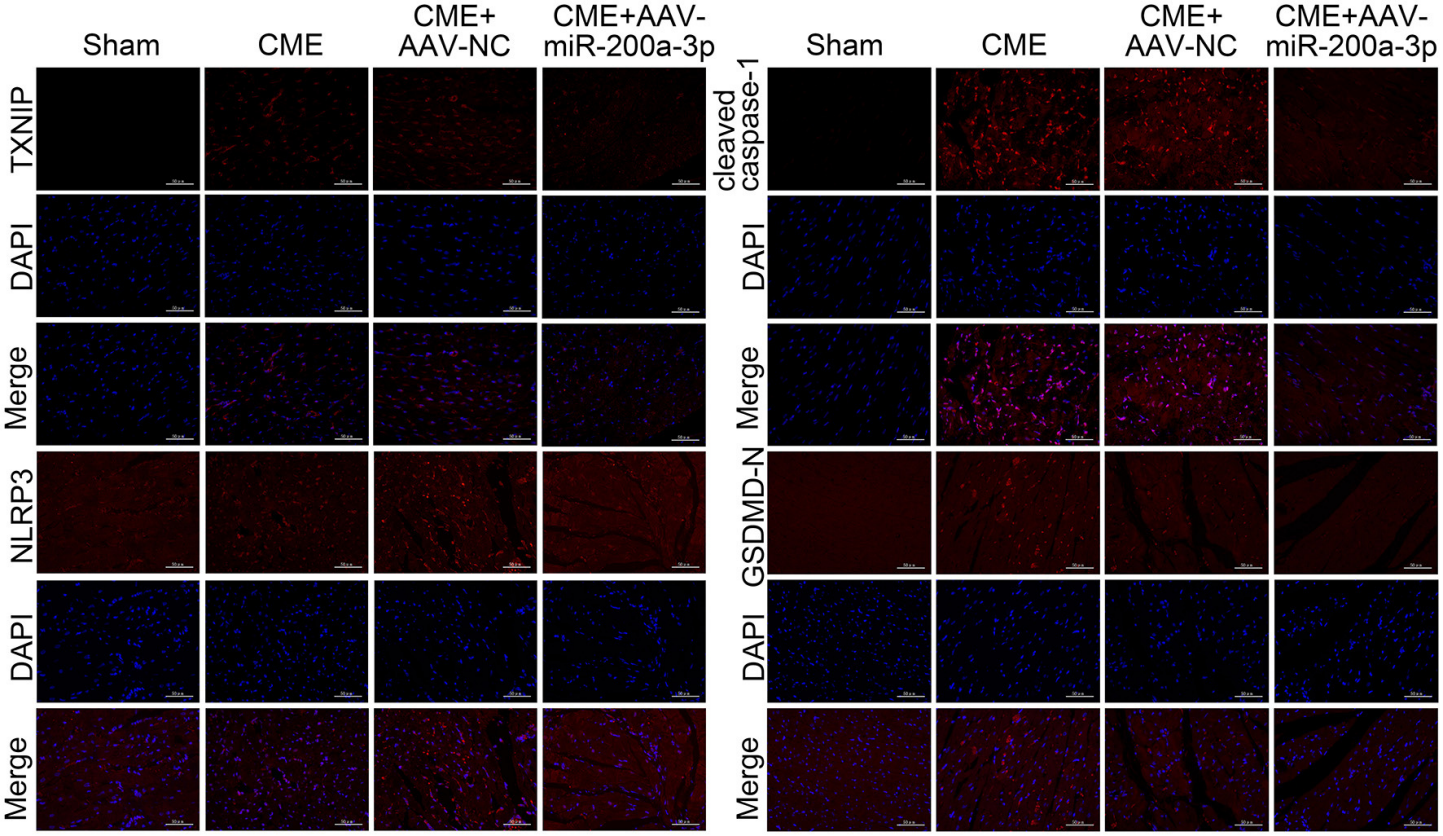
Immunofluorescence staining of TXNIP, NLRP3, cleaved caspase-1, and GSDMD-N in myocardial tissues (*n* = 3 per group). CME, coronary microembolization.

To understand how miR-200a-3p impacts CME-induced cardiomyocyte pyroptosis, we next conducted a series of experiments exploring the molecular basis for our above results. In qRT-PCR analyses we determined that the upregulation of miR-200a-3p was associated with significant reductions in NLRP3, ASC, IL-1β, and IL-18 mRNA expression relative to levels in control rats (*P* < 0.05, Figure 7A). ELISAs similarly revealed that AAV-mediated increases in miR-200a-3p expression were associated with lower serum IL-1β and IL-18 levels relative to those in rats in the CME+AAV-NC group (*P* < 0.05, Figure 7B). In Western blotting analyses, we found that AAV-miR-200-3p treatment was not associated with changes in pro-caspase-1 or GSDMD-FL expression (*P* > 0.05), whereas it was associated with significant reductions in TXNIP, NLRP3, ASC, cleaved caspase-1, GSDMD-N, IL-1β, and IL-18 protein levels (*P* < 0.05, Figure 7C). Immunofluorescence staining also confirmed that myocardial TXNIP, NLRP3, cleaved caspase-1, and GSDMD-N levels were significantly reduced in rats in the CME+AAV-miR-200a-3p group relative to the CME+AAV-NC group (Figure 8). These data together suggest that miR-200a-3p upregulation is associated with the inhibition of the TXNIP/NLRP3 signaling pathway and the consequent amelioration of CME-induced myocardial injury.

## Discussion

**Figure 9:**
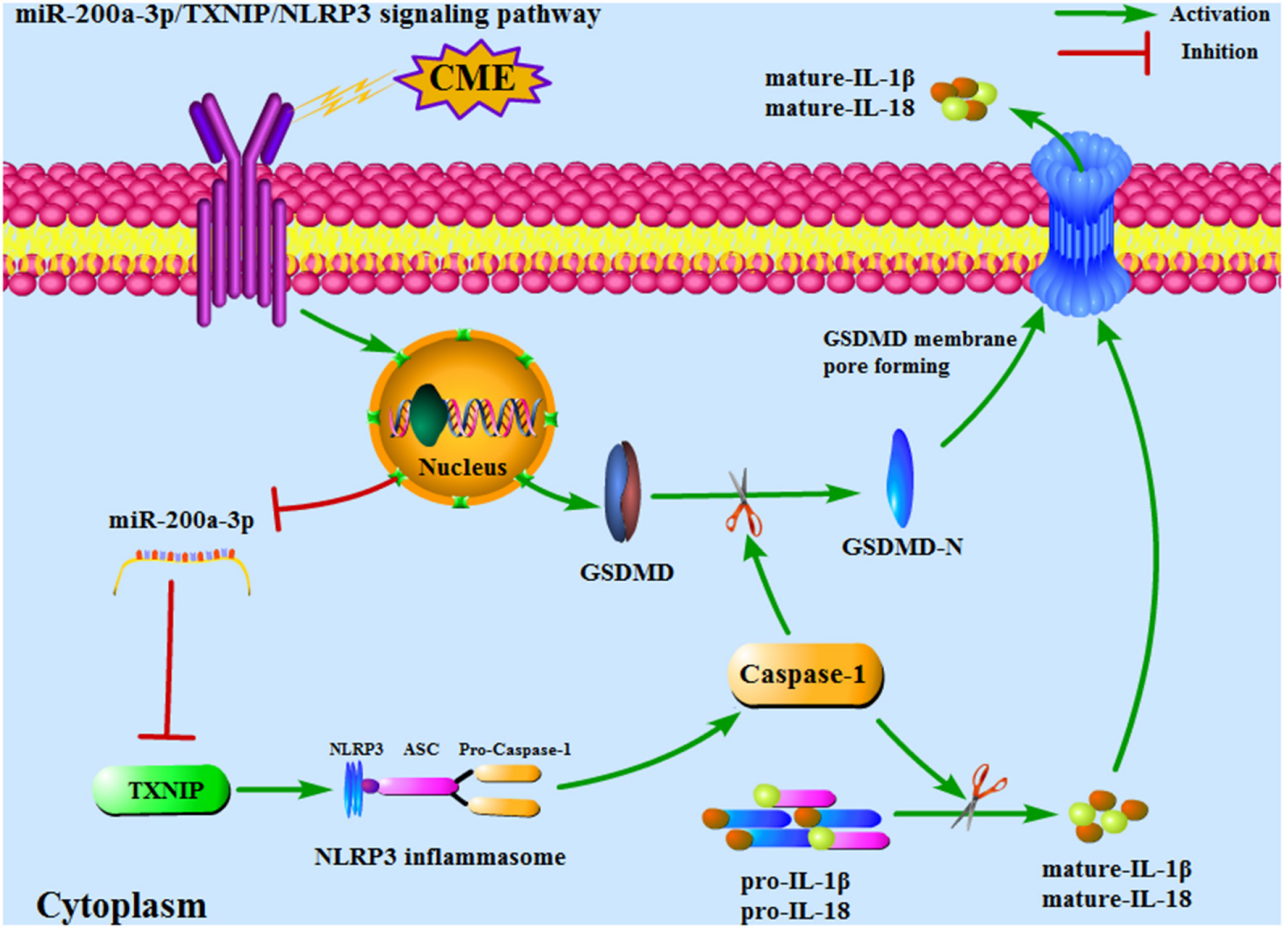
An overview of the mechanisms whereby miR-200a-3p targets the TXNIP/NLRP3 signaling pathway to modulate cardiomyocyte pyroptosis. CME, coronary microembolization.

Herein, we evaluated the role of miR-200a-3p as a regulator of CME-induced cardiomyocyte pyroptosis in rats. Through these studies, we determined that CME resulted in significant miR-200a-3p downregulation, cardiomyocyte pyroptosis, and oxidative stress, resulting in myocardial damage and cardiac dysfunction. When we used an AAV vector to upregulate miR-200a-3p in the rat model of CME, we found that this resulted in the alleviation of CME-induced pathogenesis. We additionally validated TXNIP as a target gene of miR-200a-3p using a dual-luciferase reporter assay, and confirmed that miR-200a-3p was primarily able to attenuate cardiomyocyte pyroptosis by inhibiting TXNIP/NLRP3 signaling pathway (Figure 9). Together, these findings provide new ideas for the prevention and treatment of CME-induced myocardial injury and cardiac dysfunction. However, more researches are required before these findings can be applied to clinical settings.

CME is a potential complication of PCI that can result in coronary microvascular dysfunction and heart failure (31). Pyroptosis is a form of gasdermin-mediating inflammatory programmed cell death that results in a pronounced inflammatory response that coincides with the release of intracellular components (32). We have previously demonstrated that CME can cause significant myocardial inflammation in rats, with the inhibition of this inflammation being sufficient to reduce the severity of myocardial injury and to bolster cardiac function (33). However, few studies have described the role of pyroptotic inflammation in CME-induced myocardial injury. It is of great practical significance to explore whether the inhibition of cardiomyocyte pyroptosis can reduce the inflammatory response induced by CME, thereby improving myocardial injury. In this study, we found that all pyroptotic indicators were significantly upregulated following CME, and that myocardial contractile function was markedly impaired.

Many studies have demonstrated that miRNAs can regulate a variety of pathological processes linked to cardiovascular disease by regulating inflammatory and immunological processes (34, 35). miR-200a-3p is a miR-200 family member that is known to be important in the context of myocardial ischemia-reperfusion injury (24), but it has primarily been studied in oncological contexts. How miR-200a-3p impacts CME-induced myocardial injury has not previously been reported, and studies of this regulatory relationship may thus highlight novel approaches to treating or preventing cardiac damage caused by CME. We herein found that miR-200a-3p was significantly downregulated in our rat model of CME, with such downregulation correlating with impaired cardiac function and myocardial injury, whereas miR-200a-3p upregulation was sufficient to attenuate such impairment.

NLRP3 inflammasome activation leads to the caspase-1-mediated cleavage of GSDMD to yield N-terminal fragments that subsequently induce the onset of pyroptosis (36). TXNIP is also able to bind to NLRP3 such that a lack of TXNIP can affect NLRP3 inflammasome activation and IL-1β secretion (37). This TXNIP/NLRP3 signaling pathway can drive pyroptosis, and as such, inhibiting TXNIP represents a viable approach to protecting cells against this form of programmed cell death (13, 14). Our data are consistent with those of other studies indicating that CME can induce the activation of TXNIP/NLRP3 signaling pathway and cardiomyocyte pyroptosis to aggravate myocardial injury, whereas inhibiting this pathway can protect against such pyroptosis-associated myocardial injury.

Through a dual-luciferase reporter assay, we determined that miR-200-3p was able to directly target and suppress TXNIP expression. We then upregulated the expression of miR-200a-3p by transfecting AAV-miR-200a-3p into rats to verify the interaction between miR-200a-3p and TXNIP and the effect of miR-200a-3p/TXNIP/NLRP3 signaling pathway on cardiomyocyte pyroptosis in the context of CME. These results indicate that miR-200a-3p overexpression can inhibit the TXNIP/NLRP3 signaling pathway to suppress pyroptosis and subsequent myocardial injury caused by CME, confirming the importance of the miR-200a-3p/TXNIP/NLRP3 signaling axis in the induction of cardiomyocyte pyroptosis.

There are some limitations in this study. First, our CME model was established through the direct injection of plastic microspheres into coronary microcirculation, and these spheres will result in pathological changes distinct from those caused by atherosclerotic plaques in clinical settings. Indeed, clinical emboli can be complex and exhibit a range of biological properties not recapitulated by these microspheres. Second, it is unavoidable that other forms of cardiomyocyte death may also participate in CME-induced myocardial injury, such as apoptosis, necroptosis and necrosis. The extent to which each form of cell death causes myocardial damage remains to be further studied. Third, pyroptosis is a multifaceted complex pathway, and we therefore cannot exclude the possibility that CME-induced myocardial injury is influenced by other signaling pathways not evaluated herein. Further research will thus be required to address these limitations and to expand upon our present findings.

In summary, we herein determined that miR-200a-3p can inhibit cardiomyocyte pyroptosis by suppressing the TXNIP/NLRP3 signaling pathway, thus alleviating CME-induced myocardial injury and restoring normal cardiac functionality. These results highlight a novel approach to the potential treatment or prevention of myocardial injury induced by CME.

## Data Availability Statement

The raw data supporting the conclusions of this article will be made available by the authors, without undue reservation.

## Ethics Statement

The animal study was reviewed and approved by The Animal Use Ethics Committee of Guangxi Medical University.

## Author Contributions

Z-QC and YZ conceived and designed research. Z-QC, FC, and J-WH conducted experiments. H-LL and TL collected and analyzed the data. Z-QC wrote the manuscript. YZ and LL reviewed and revised the manuscript. All authors read and approved the manuscript.

## Conflict of Interest

The authors declare that the research was conducted in the absence of any commercial or financial relationships that could be construed as a potential conflict of interest.

## Publisher's Note

All claims expressed in this article are solely those of the authors and do not necessarily represent those of their affiliated organizations, or those of the publisher, the editors and the reviewers. Any product that may be evaluated in this article, or claim that may be made by its manufacturer, is not guaranteed or endorsed by the publisher.
